# Production of Monodisperse Oil-in-Water Droplets and Polymeric Microspheres Below 20 μm Using a PDMS-Based Step Emulsification Device

**DOI:** 10.3390/mi16020132

**Published:** 2025-01-24

**Authors:** Naotomo Tottori, Seungman Choi, Takasi Nisisako

**Affiliations:** 1 Department of Mechanical Engineering, School of Engineering, Institute of Science Tokyo, Tokyo 152-8550, Japan; 2 Laboratory for Future Interdisciplinary Research of Science and Technology (FIRST), Institute of Integrated Research, Institute of Science Tokyo, R2-9, 4259 Nagatsuta-cho, Midori-ku, Yokohama 226-8501, Japan

**Keywords:** microfluidics, step emulsification, PDMS, oil-in-water droplets, polymeric microspheres

## Abstract

Step emulsification (SE) is renowned for its robustness in generating monodisperse emulsion droplets at arrayed nozzles. However, few studies have explored poly(dimethylsiloxane) (PDMS)-based SE devices for producing monodisperse oil-in-water (O/W) droplets and polymeric microspheres with diameters below 20 µm—materials with broad applicability. In this study, we present a PDMS-based microfluidic SE device designed to achieve this goal. Two devices with 264 nozzles each were fabricated, featuring straight and triangular nozzle configurations, both with a height of 4 µm and a minimum width of 10 µm. The devices were rendered hydrophilic via oxygen plasma treatment. A photocurable acrylate monomer served as the dispersed phase, while an aqueous polyvinyl alcohol solution acted as the continuous phase. The straight nozzles produced polydisperse droplets with diameters exceeding 30 µm and coefficient-of-variation (CV) values above 10%. In contrast, the triangular nozzles, with an opening width of 38 µm, consistently generated monodisperse droplets with diameters below 20 µm, CVs below 4%, and a maximum throughput of 0.5 mL h^−1^. Off-chip photopolymerization of these droplets yielded monodisperse acrylic microspheres. The low-cost, disposable, and scalable PDMS-based SE device offers significant potential for applications spanning from laboratory-scale research to industrial-scale particle manufacturing.

## 1. Introduction

Microfluidic technologies capable of generating uniformly sized droplets have garnered significant attention over the past two decades, with applications spanning diverse fields such as biology and materials science [1]. A notable example is droplet-based digital polymerase chain reaction (dPCR), which has seen widespread adoption in biological applications [2]. Similarly, droplet-generating microfluidic technologies have been employed for producing functional particles, typically through the solidification of monodisperse precursor droplets generated by various microfluidic devices [3].

Among the various microfluidic droplet-generating technologies, step emulsification (SE), originally referred to as ‘microchannel emulsification’ [4], has gained considerable attention in recent years, largely due to its robustness in parallelization [5,6]. SE devices, along with their variants [7,8], are characterized by shallow microfabricated nozzles leading to a deeper cavity, where uniformly sized droplets are generated one by one in the continuous phase. Unlike droplet formation driven by viscous shear forces, SE nozzles enable spontaneous droplet break-off via an interfacial-tension-driven mechanism. The Laplace pressure difference between the forming droplet in the deeper cavity and the dispersed phase on the shallow nozzle induces localized fluid flow and necking, leading to droplet snap-off through interfacial instability. Droplet size is heavily influenced by the wetting properties [9] and geometry [10] of the nozzles, particularly nozzle height. Precise microfabrication of arrayed nozzles ensures droplet monodispersity across the array, making SE devices highly suitable for parallelization and production scaling.

To date, a variety of materials have been employed in fabricating SE devices. Earlier devices were commonly fabricated using wet or dry etching of silicon substrates [4,11], while rigid materials such as glass [8,9,12], stainless steel [13], and PMMA [14,15,16] have also been utilized. These devices have demonstrated the ability to produce monodisperse droplets with diameters ranging from sub-micrometer to millimeter scales [5,6]. More recently, poly(dimethylsiloxane) (PDMS)—a gold standard in soft lithography for microfluidic device development [17]—has been increasingly used in SE device fabrication, primarily due to its ease of fabrication, cost-effective replication, and suitability for disposable applications in laboratory settings. Confined SE nozzle arrays are prone to clogging, particularly during prolonged continuous operation. In cases where cleaning is challenging, as with permanently bonded devices, low-cost, disposable devices are highly advantageous.

Most studies on PDMS-based SE devices have focused on generating water-in-oil (W/O) droplets [10,18,19,20,21,22,23,24,25], driven by the widespread demand for aqueous droplets in bioapplications and the intrinsic hydrophobicity of PDMS. While most of these studies produced W/O droplets larger than 20 µm, Shin et al. achieved a minimum droplet size of 17 µm using a centrifuge-based SE device with a 4 µm high nozzle [22]. In contrast, research on PDMS-based SE devices for oil-in-water (O/W) droplet production, particularly for particle synthesis, is limited. Mittal et al. [26] demonstrated the production of O/W droplets with a minimum diameter below 9 µm by dispersing fluorinated oils, mineral oil, or silicone oils in aqueous sodium dodecyl sulfate solutions using a PDMS-glass SE device with 1000 parallel straight nozzles. Nalin et al. [27] employed pneumatically actuated hydrophilic PDMS-based SE nozzles to generate hexadecane droplets with diameters exceeding 50 µm in aqueous biopolymer solutions for 3D-printed porous materials. Additionally, hydrophilic PDMS-based SE devices have been applied as a second step in tandem water-in-oil-in-water (W/O/W) double emulsification processes [28,29]. However, studies specifically focusing on particle synthesis from O/W droplets using PDMS-based SE devices remain scarce.

In our previous work, we demonstrated the production of monodisperse droplets of an acrylate monomer in aqueous polyvinyl alcohol (PVA) solutions to synthesize monodisperse polymeric microspheres with diameters exceeding 50 µm using PDMS–glass [30] and PDMS–stainless steel [31] SE devices equipped with triangular nozzles with a height of 16 µm. However, the production of smaller monodisperse microspheres is highly desirable for a broader range of applications requiring higher surface-to-volume ratios, such as drug delivery systems, immunoassays, liquid chromatography, advanced coatings, and optical materials [32]. Despite their potential, the synthesis of such microspheres using disposable and scalable PDMS-based SE devices remains largely underexplored.

In this study, we demonstrate the production of monodisperse O/W droplets and polymeric microspheres with average diameters below 20 µm and CVs below 4% using a PDMS-based SE device. Two SE devices with different nozzle configurations—straight nozzles and triangular nozzles—were fabricated, both with a minimum width of 10 µm and a height of 4 µm, which is one-quarter the height of the nozzles used in our previous devices [30,31]. The devices were rendered hydrophilic prior to use to facilitate O/W droplet formation. Their performance was evaluated based on the sizes and size distributions of droplets generated under varying flow rates. The monodisperse O/W droplets generated by the triangular SE nozzles were subsequently photopolymerized to yield monodisperse polymeric microspheres.

## 2. Materials and Methods

### 2.1. Device Design

**Figure 1:**
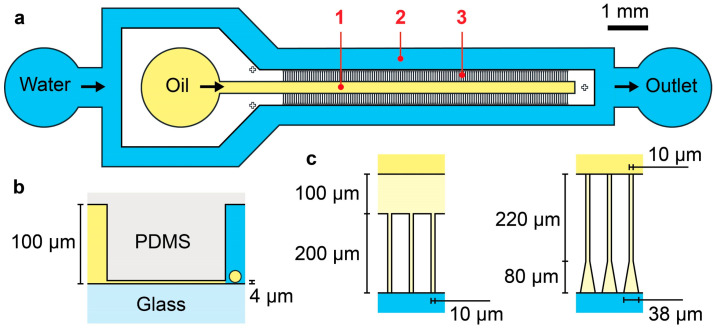
Polydimethylsiloxane (PDMS)-based step-emulsification (SE) devices for generating oil-in-water (O/W) droplets with diameters below 20 µm. (**a**) Schematic representation of the overall channel layout, showing: (1) a central channel for introducing the dispersed oil phase, (2) two side channels for supplying the continuous aqueous phase and collecting the produced droplets, and (3) two arrays of 132 shallow SE nozzles (264 nozzles in total). (**b**) Schematic cross-sectional view of the nozzle and channels, highlighting their respective heights. (**c**) Top-view illustrations of nozzle configurations, depicting straight nozzles with an upstream plateau (left) and triangular nozzles (right) along with their geometric parameters.

Figure 1a illustrates the basic channel layout of the SE devices utilized in this study, featuring a “millipede” configuration [10]. This design consists of a deep central channel (width: 0.3 mm) for introducing the dispersed organic phase, two deep side channels (width: 0.5 mm) for infusing the continuous aqueous phase and collecting the droplets, and two parallel arrays of 132 shallow SE nozzles (264 nozzles in total) connecting the central and side channels. The central and side channels have a depth of 100 µm, while the SE nozzles are 4 µm deep (Figure 1b). All channels have rectangular cross-sections.

To evaluate droplet generation performance, we fabricated two SE devices with distinct nozzle configurations: straight nozzles and triangular nozzles (Figure 1c). The straight nozzles were arrayed with a pitch of 55 µm and featured an upstream plateau of 100 µm long, followed by a downstream channel (10 µm wide and 200 µm long) leading to the step (Figure S1). The triangular nozzles were also arrayed with a pitch of 55 µm, incorporating an upstream straight channel (10 µm wide and 220 µm long) that gradually broadened to 38 µm along its 80 µm length toward the downstream step.

### 2.2. Microfabrication

**Figure 2:**
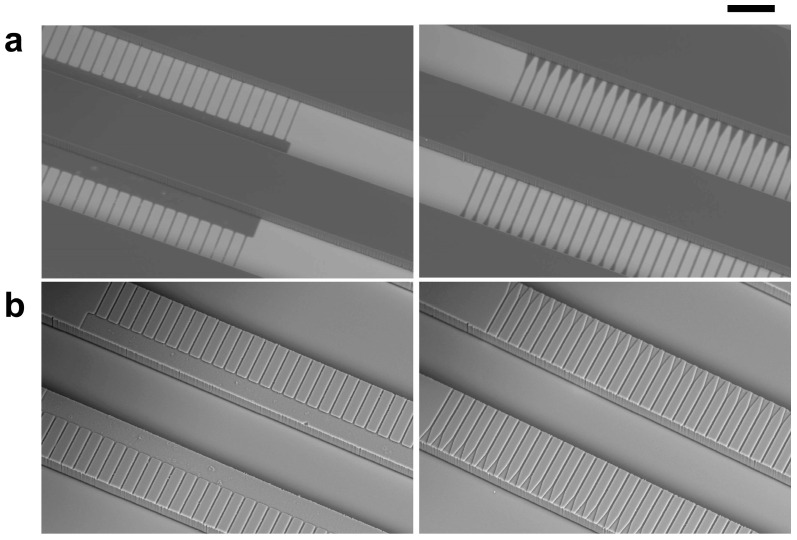
Scanning electron microscopy (SEM) images of (**a**) master molds and (**b**) microchannels replicated in PDMS chips for the SE devices with straight nozzles (**left**) and triangular nozzles (**right**). Scale bar: 200 μm.

The two SE devices were fabricated from PDMS using a standard soft lithography technique. Master molds for each of the devices, incorporating two layers for the shallow nozzles and deep peripheral channels, were prepared via two-step photolithography, as described previously [30]. In the first step, a thin layer for the nozzles (thickness: 4 μm) was created by patterning the negative photoresist SU-8 3005 (Kayaku Advanced Materials, MA, USA) on a silicon wafer. In the second step, a thick layer for the peripheral channels (thickness: 100 μm) was fabricated on top of the nozzle layer using SU-8 3050 (Kayaku Advanced Materials). For the nozzle and peripheral channel patterns, two separate photomask films printed at 12,700 dpi (UnnoGiken, Tokyo, Japan) were used. Scanning electron microscopy (SEM) images of the molds for straight nozzles and triangular nozzles are shown in Figure 2a and Figure S2a.

The master molds were treated with 0.5 mL of vaporized trimethylchlorosilane (C0306; Tokyo Chemical Industry, Tokyo, Japan) inside a disposable Petri dish to promote mold release. A PDMS prepolymer (Silpot 184 w/c; Dow Toray, Tokyo, Japan), mixed with a catalyst at a 10:1 *w*/*w* ratio, was degassed in a vacuum desiccator, poured onto the master mold, and cured on a hot plate at 80 °C for 1 h. Once cured, the crosslinked PDMS was carefully peeled off the mold (Figure 2b and Figure S2b), and inlet and outlet holes (1.0 mm in diameter) were punched using a precision punch tool (BP-10F; Kai Industry, Gifu, Japan).

The PDMS component was then irreversibly sealed with a glass slide (S1111; Matsunami Glass, Tokyo, Japan) through oxygen plasma treatment (BP-1; Samco, Tokyo, Japan) for 30 s at 20 W, followed by baking on a hot plate at 80 °C for 10 min. Before use, the devices underwent an additional 10 min oxygen plasma treatment at 20 W to render their channel surfaces hydrophilic. To preserve hydrophilicity, the treated devices were immediately immersed in deionized water and utilized within a few hours.

### 2.3. Chemicals

To generate oil droplets dispersed in an aqueous solution, the organic phase consisted of 1,6-hexanediol diacrylate (HDDA) (A-HD-N; density: 1.02 g/cm^3^; dynamic viscosity: 6.35 mPa s; Shin-Nakamura Chemical, Wakayama, Japan) with 3 wt% photoinitiator (Darocur 1173; BASF Japan, Tokyo, Japan). The aqueous phase was prepared as a 2.0 wt% solution of PVA (GL-03; *M*_w_~20,000 g/mol; degree of hydrolysis: 87–89%; Mitsubishi Chemical, Tokyo, Japan) dissolved in deionized water (Direct-Q UV3; Merck, Hessen, Germany). For the experiment involving aqueous droplets in oil, deionized water was used as the dispersed phase, and corn oil (Fujifilm Wako Pure Chemicals, Tokyo, Japan) containing 1 wt% surfactant (CRS-75; Sakamoto Yakuhin Kogyo, Osaka, Japan) was prepared as the continuous phase. Acetone and ethanol (Fujifilm Wako Pure Chemicals) were used for washing the photopolymerized microspheres.

### 2.4. Preparation of Polymeric Microspheres

Photocurable monomer droplets exiting the SE devices were collected and subjected to UV irradiation (LA-410UV, Hayashi-repic, Tokyo, Japan) from a distance of 10–15 cm for 30 s to induce photopolymerization. The resulting polymeric microspheres were characterized using optical microscopy, offering a maximum resolution of 0.5 µm per pixel, and low-vacuum SEM at 30 kV and 45 Pa (JSM-6610LA; JEOL, Tokyo, Japan).

### 2.5. General Equipment

Two gastight glass syringes (1 mL; Hamilton Company, NV, USA) containing the respective liquids were connected to the SE device inlets via polyethylene tubes (internal diameter: 0.5 mm; external diameter: 1.0 mm; Hibiki #3; Kunii, Tokyo, Japan). The syringes were mounted on syringe pumps (KDS200; KD Scientific, MA, USA) to ensure precise infusion of the liquids into the microchannels. Droplet formation at the SE nozzles was monitored and recorded using an inverted optical microscope (CKX41; Evident, Tokyo, Japan) equipped with a high-speed camera (FASTCAM Mini AX50; Photron, Tokyo, Japan).

## 3. Results

### 3.1. Straight Nozzles

We evaluated the performance of the SE device with straight nozzles to assess its capability for generating monodisperse O/W droplets with an average diameter below 20 µm. To prepare the device for droplet formation, the two side channels were first filled with the aqueous PVA solution via the water inlet. Subsequently, HDDA was infused through the oil inlet, displacing air in the central channel, which was expelled as bubbles through the nozzles to the outlet. Once all air bubbles were eliminated and nozzles were fully filled with HDDA, droplet formation experiments were initiated by adjusting the flow rates using syringe pumps.

**Figure 3:**
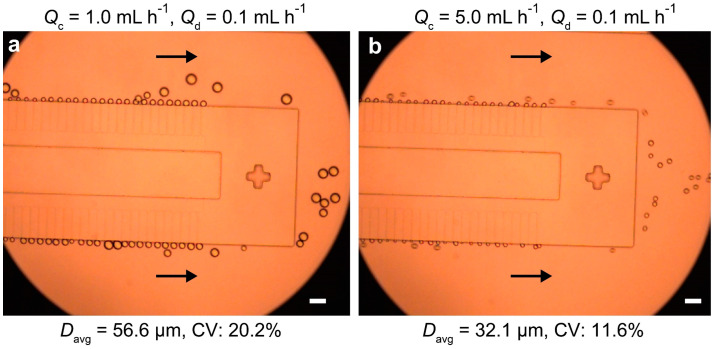
Generation of polydisperse O/W droplets in the straight-nozzle device. (**a**) Droplet formation at a dispersed phase flow rate (*Q*_d_) of 0.1 mL h^−1^ and a continuous phase flow rate (*Q*_c_) of 1.0 mL h^−1^. (**b**) Droplet formation under the same *Q*_d_ but at *Q*_c_ = 5.0 mL h^−1^. Arrows indicate the continuous phase flow direction. Scale bars: 100 μm.

Figure 3a shows the downstream nozzle region and the resulting droplets when the continuous phase (*Q*_c_) and dispersed phase (*Q*_d_) flow rates were set at 1.0 mL h^−1^ and 0.1 mL h^−1^, respectively. O/W droplets were formed at nearly all nozzle exits; however, their sizes were significantly larger than the nozzle width (10 µm) and exhibited considerable non-uniformity, with an average diameter (*D*_avg_) of 57 µm and a coefficient of variation (CV) of 20.2% (*n* = 17). Despite varying *Q*_d_ in the range from 0.01 to 1.0 mL h^−1^, the droplet sizes remained larger than 20 µm and were polydisperse. Increasing *Q*_c_ from 1.0 to 5.0 mL h^−1^ reduced both *D*_avg_ and CV to 32 µm and 11.6%, respectively (*n* = 22, Figure 3b). Nevertheless, the droplets were still larger than 20 µm and polydisperse. Further increasing *Q*_c_ to 10.0 mL h^−1^ nearly deactivated the upstream nozzles.

The observed reduction in droplet size with increasing *Q*_c_ suggests that droplet breakup was influenced by viscous shearing from the cross-flowing aqueous stream. This behavior indicates that the droplets were generated in the so-called ‘burst’ or ‘large drop’ (LD) mode, where droplet formation is no longer spontaneous. The LD mode typically produces larger droplets compared to the ‘small drop’ (SD) mode, which is entirely driven by interfacial tension. It has been reported that in the LD mode, droplet collisions with forming droplets can induce irregular breakup [26]. Additionally, droplets flowing near the nozzles may create uneven shear forces on forming droplets, further contributing to the wider size distribution observed in this study.

### 3.2. Triangular Nozzles

**Figure 4:**
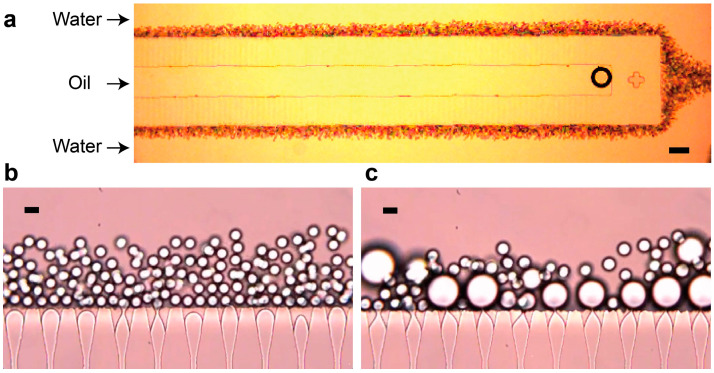
O/W droplet generation using the device with triangular nozzles. (**a**) Optical micrograph of the nozzle arrays operating at *Q*_c_ = 5.0 mL h^−1^ and *Q*_d_ = 0.1 mL h^−1^. Scale bar: 200 µm. (**b**) Magnified views of the nozzles in (**a**), demonstrating operation in the ‘small drop’ (SD) mode. (**c**) Magnified view of the nozzles operating at *Q*_c_ = 5.0 mL h^−1^ and *Q*_d_ = 0.7 mL h^−1^, showing two nozzles on the left operating in the SD mode, while the remaining nozzles operate in the ‘large drop’ (LD) mode. Scale bars: 20 µm.

Next, we conducted experiments using the SE device with triangular nozzles. Similar to the device with straight nozzles, the working range of *Q*_c_ to prevent upstream nozzle deactivation was below 10.0 mL h^−1^. Figure 4a shows the operation of the nozzle array at flow rates of *Q*_c_ = 5.0 mL h^−1^ and *Q*_d_ = 0.1 mL h^−1^. Almost all of the 264 nozzles were functional, with only one nozzle clogged (99.6% operational efficiency). These droplets displayed uniform size, with diameters smaller than the nozzle width.

High-speed video observations revealed a consistent droplet formation sequence at each nozzle (Figure 4b and Video S1). (1) Following the break-off of the preceding droplet, a tongue-shaped oil–water interface forms in the triangular region. (2) The interface advances to the edge of the nozzle, forming a growing droplet and a neck at the nozzle exit. (3) As the droplet grows, the neck rapidly narrows until it breaks, releasing a discrete droplet. This reproducible behavior corresponds to the typical SD regime in step emulsification, driven primarily by the gradient of Laplace pressure (interfacial-tension-driven).

To assess flow uniformity across the nozzle array, we measured the average frequency of droplet break-off at 10 nozzles located in upstream and downstream regions. The frequencies were 30.6 ± 1.6 Hz (*n* = 10) for upstream nozzles and 31.1 ± 0.9 Hz (*n* = 10) for downstream nozzles, indicating relatively uniform dispersed phase flow rates per nozzle, despite the pressure gradient generated by the cross-flowing aqueous stream.

**Figure 5:**
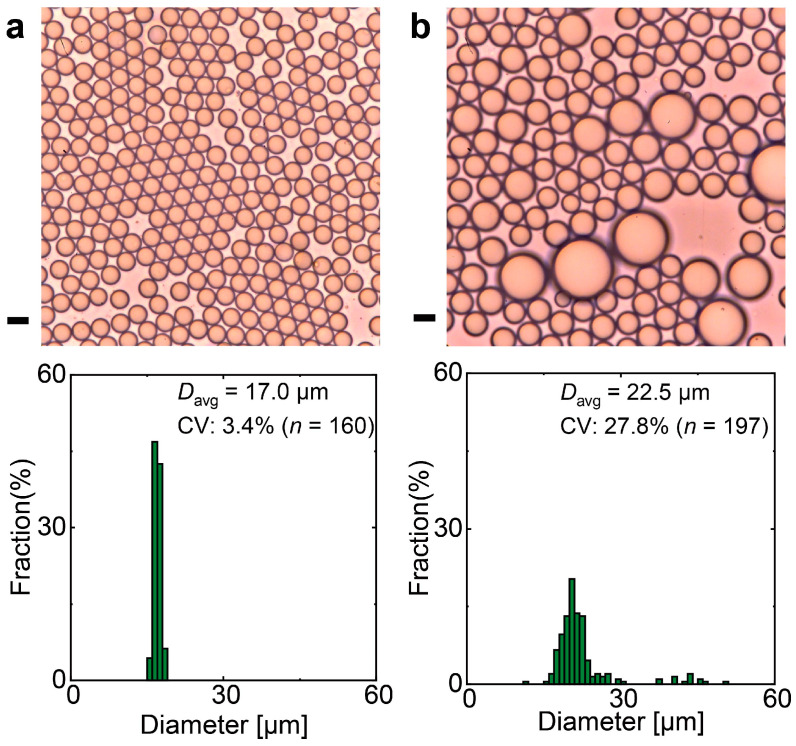
Micrographs and size distributions of O/W droplets collected from the device with triangular nozzles. Droplets were produced at *Q*_c_ = 5.0 mL h^−1^ and *Q*_d_ = (**a**) 0.1 mL h^−1^, (**b**) 0.7 mL h^−1^. Scale bars: 20 μm.

Droplets produced at *Q*_c_ = 5.0 mL h^−1^ and *Q*_d_ = 0.1 mL h^−1^ were collected and analyzed using optical microscopy. As shown in Figure 5a, the droplets were highly monodisperse, with *D*_avg_ = 17 µm and a CV of 3.4% (*n* = 160). Additionally, the droplets exhibited excellent stability against coalescence due to the presence of PVA in the continuous aqueous phase.

However, increasing *Q*_d_ to 0.7 mL h^−1^ led to the production of significantly larger droplets at majority of nozzles, similar to the behavior observed in the straight-nozzle device, while some nozzles remain to produce smaller droplets (Figure 4c and Video S2). This transition suggests a shift from the SD regime to the LD regime. The resulting droplets were highly polydisperse, with *D*_avg_ = 23 µm and a CV of 28% (*n* = 197, Figure 5b).

**Figure 6:**
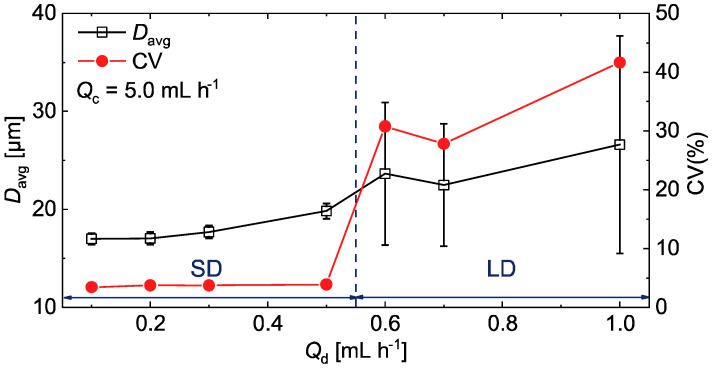
Evolution of the average droplet diameter (*D*_avg_) and CV values across the SD and LD regimes for O/W droplets generated using the device with triangular nozzles, with *Q*_d_ varied from 0.1 to 1.0 mL h^−1^ and *Q*_c_ fixed at 5.0 mL h^−1^.

To further investigate the flow conditions under which the SD-to-LD transition occurs, we varied *Q*_d_ from 0.1 to 1.0 mL h^−1^ while maintaining *Q*_c_ at 5.0 mL h^−1^. As *Q*_d_ was gradually increased from 0.1 mL h^−1^, droplets remained consistently monodisperse, with CV values below 4%. The *D*_avg_ increased slightly but remained below 20 µm up to *Q*_d_ = 0.5 mL h^−1^ (Figure 6 and Figure S3a,b), indicating that the nozzles operated within the SD regime. However, at *Q*_d_ = 0.6 mL h^−1^, some nozzles began producing significantly larger droplets, signaling a transition to the LD mode. This resulted in polydisperse droplets, with an abrupt increase in *D*_avg_ exceeding 20 µm and a sharp rise in CV to approximately 30% (Figure S3c). At *Q*_d_ = 1.0 mL h^−1^, the CV increased further, exceeding 40% (Figure S3d). These findings suggest that the critical *Q*_d_ for the transition from SD to LD lies between 0.5 and 0.6 mL h^−1^. Consequently, we confirmed that monodisperse O/W droplets with *D*_avg_ below 20 µm could be reliably generated at *Q*_d_ values up to 0.5 mL h^−1^ in the SE device with separated triangular nozzles.

**Figure 7:**
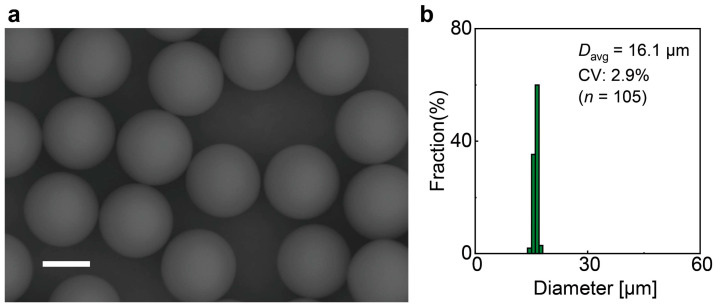
Monodisperse polymeric microspheres obtained through off-chip photopolymerization. (**a**) SEM images of polymeric microspheres derived from O/W droplets generated by the device with edge-shaped nozzles at *Q*_c_ = 5.0 mL h^−1^ and *Q*_d_ = 0.1 mL h^−1^. (**b**) Size distribution of the microspheres. Scale bar: 10 µm.

Irradiating UV light onto the monodisperse droplets exiting the device resulted in the formation of monodisperse polymeric microspheres (Figure 7a). For example, at *Q*_c_ = 5.0 mL h^−1^ and *Q*_d_ = 0.1 mL h^−1^, the produced particles had *D*_avg_ = 16 µm with a CV of 2.9% (*n* = 105, Figure 7b). The polymerized particles were approximately 6% smaller than their precursor droplets before photopolymerization. Particles produced at a higher *Q*_d_ value also exhibited a similar shrinkage percentage compared to their precursor droplets (Figure S4), consistent with findings from similar experiments using the same acrylate monomer [30,31] (Table S1). Although particle shrinkage is a well-known phenomenon associated with photocurable materials, thermosetting materials [33], which typically exhibit lower shrinkage, represent a promising alternative to mitigate this issue and enhance particle size consistency, making them particularly suitable for applications requiring high precision.

## 4. Discussion

### 4.1. Straight Nozzles

Previous studies reported monodisperse O/W droplet generation in the SD mode using straight SE nozzles with varying nozzle width (*w*) and height (*h*). Mittal et al. demonstrated the production of O/W droplets with diameters of 8–10 µm, approximately four times the nozzle height, using PDMS-based straight nozzles with *w* = 10 µm and *h* = 2 µm (*w*/*h* = 5), rendered hydrophilic via oxygen plasma treatment [26]. Similarly, Sahin et al. achieved droplets of comparable sizes using glass straight nozzles with *w* = 5 µm and *h* = 2 µm (*w*/*h* = 2.5) [8].

In contrast, our PDMS-based straight nozzles with *w*/*h* = 2.5 produced droplets with average diameters significantly exceeding 4*h*. Based on these reports, two modifications may effectively improve the performance of our straight nozzles. First, increasing the nozzle width to achieve a higher *w*/*h* aspect ratio, such as *w* = 20 µm and *w*/*h* = 5, could enhance interfacial instability, promoting efficient droplet snap-off. Second, replacing the oxygen plasma treatment with alternative surface modification methods may yield more reliable hydrophilic nozzle surfaces, as discussed in the following section.

### 4.2. Triangular Nozzles

For PDMS-based SE devices with triangular nozzles, Amstad et al. reported an optimized range of nozzle width *w* and height *h* ratios for monodisperse W/O droplet generation as 5.5 < *w*/*h* < 19, with droplet diameter increasing as *w* increases [10]. Our PDMS-based nozzles for O/W droplet generation have *w*/*h* = 38/4 = 9.5, which falls within this range. In previous experiments using the same fluids as the present study, O/W droplets were generated in PDMS-stainless steel devices with *w*/*h* = 6.8 [31] and in PDMS-glass devices with *w*/*h* = 7.8 [30], producing droplets with diameters of 4.0–4.3 *h* and 3.7–3.8 *h*, respectively. In the present study, the droplet diameters ranged from 4.25 to 5 *h*, slightly larger than the coefficients observed in previous results.

We attribute these slightly larger coefficients to the larger *w*/*h* ratio of the triangular nozzles used in the present study (*w*/*h* = 9.5) compared to the previous devices (*w*/*h* = 6.8 and 7.8). Additionally, differences in the hydrophilic surface modification techniques applied to the devices may have contributed to the observed variation.

We expect the current nozzles to produce droplets of similar sizes with different liquid combinations. However, variations in fluid viscosities and interfacial tensions may result in slight differences in droplet size within the same device.

### 4.3. Advantages, Limitations, and Scope of the PDMS-Based SE Device

We conducted experiments using two PDMS-based SE devices with distinct nozzle configurations, straight and triangular nozzles, with the same height (4 µm) and minimum width (10 µm) and rendered hydrophilic through oxygen-plasma surface modification. The device with straight nozzles, which had a smaller opening (10 µm × 4 µm), was unable to operate in the interfacial-tension-driven SD mode, instead producing polydisperse O/W droplets in the shear-driven LD mode, with average diameters significantly exceeding 20 µm. In contrast, the triangular nozzles with larger opening (38 µm × 4 µm) successfully operated in the SD mode, generating monodisperse O/W droplets with average diameters below 20 µm and CVs below 4%.

The hydrophilic surface modification achieved through oxygen plasma treatment has both advantages and limitations. A key advantage is its reusability: if the hydrophilicity degrades over time during experiments, oxygen plasma treatment can be reapplied to the dried device to restore the hydrophilic surface of the internal nozzles. Additionally, the hydrophilic PDMS surface can be reverted to a hydrophobic state by baking, enabling the device to produce water-in-oil (W/O) droplets (Figure S5). However, a notable limitation of this treatment is its relatively short effective lifetime. In our experiments, we observed partial wetting of HDDA on the nozzles, likely due to the degradation of hydrophilicity within a few hours (Figure S6). Furthermore, oxygen plasma treatment requires specialized equipment, which may limit accessibility for some users. For extended operations, replacing oxygen plasma treatment with permanent hydrophilic coatings—such as introducing aqueous solutions of polyelectrolytes [34], PVA [35], or superhydrophilic polymer [30,31,36] into the devices—may be preferable.

Polymeric microspheres with diameters ranging from 10 to 20 µm offer an optimal balance between small size and ease of handling, making them highly versatile for various applications. Further reduction in the size of O/W droplets and the resulting polymeric microspheres could be achieved by using PDMS nozzles with smaller dimensions. The SE device with triangular nozzles of 4 µm height produced O/W droplets with diameters in the range of 17–20 µm, corresponding to a *D*_avg_/*h* ratio of 4.25–5. Since droplet size is approximately proportional to nozzle height, reducing *h* to 2 µm—half the current nozzle height—could result in droplets with diameters below 10 µm. For droplets below 5 µm, nozzles with *h* = 1 µm would be required. The fabrication of such nozzles is feasible using conventional photolithography to create master molds, followed by PDMS replication. However, achieving hydrophilic modification in such small dimensions may be challenging due to increased hydraulic resistance and the difficulty of plasma treatment. In this case, the use of hydrophilic PDMS prepolymers for casting [37] could be a promising alternative.

Another potential limitation is the compatibility of PDMS with organic solvents. While no significant swelling of PDMS due to HDDA absorption was observed during our experiments, it is well documented that PDMS exhibits varying degrees of compatibility with different organic solvents [38]. Severe absorption of an organic solvent by PDMS can lead to considerable swelling of the channel walls, resulting in channel narrowing or even clogging—issues that become particularly critical in shallow nozzles with smaller dimensions. However, when the organic dispersed phase is compatible with PDMS, as demonstrated with HDDA in this study, SE devices fabricated from PDMS remain a promising option for generating monodisperse O/W droplets. Furthermore, applying hydrophilic, solvent-resistant coatings, such as gelatin-methacryloyl polymer coatings [27] or hybrid inorganic/organic polymer (HR4) coatings [39], could further enhance PDMS compatibility with a broader range of organic solvents.

The maximum throughput of monodisperse O/W droplets achieved in this study was 0.5 mL h^−1^, equivalent to approximately 3.0 × 10^9^ droplets per device, 1.1 × 10^7^ droplets per nozzle, and 12 g of product per day (24 h). To further scale up production throughput, a combination of three approaches—array extension, array parallelization, and modular parallelization—holds significant promise. First, the nozzle array in our device can be further extended to include additional nozzles until the upstream nozzles are deactivated by the increased pressure generated by the cross-flowing continuous phase. For higher-density parallelization of the nozzles, it will be important to determine the minimum *w*/*h* ratio capable of producing monodisperse O/W droplets. Second, as demonstrated in our recent work [36], at least 10 sets of the millipede design can be parallelized within the footprint of a 76 × 52 mm glass slide without compromising the monodispersity of the produced droplets. Finally, operating more than 10 such modules in parallel [40] would enable a scaling factor exceeding 100-fold, corresponding to a production throughput on the order of kilograms per day.

We believe it is evident that soft PDMS chips are significantly more cost-effective to fabricate in a standard laboratory setting, with costs of less than USD 10 per chip (excluding equipment setup costs), compared to hard silicon or glass chips, which require wet/dry etching processes in cleanroom facilities. This is particularly advantageous during the development stage. However, for industrial-scale mass production, alternative methods such as using low-cost engineering plastics like polycarbonate (PC) and PMMA combined with injection molding may offer better cost efficiency, provided that nozzle heights below several micrometers can be reliably fabricated with high precision.

## 5. Conclusions

We demonstrated the generation of O/W emulsion droplets using two SE devices fabricated from PDMS, each equipped with 264 nozzles of 4 µm height and distinct configurations. While the straight nozzles produced O/W droplets with diameters significantly exceeding 20 µm in a shear-driven mode, the triangular nozzles reliably generated monodisperse O/W droplets in an interfacial-tension-driven mode. These droplets had average diameters below 20 µm and CVs below 4%, with a maximum throughput of 0.5 mL h^−1^. Subsequent off-chip photopolymerization successfully yielded monodisperse polymeric microspheres.

The proposed SE devices are cost-effective to fabricate, making them suitable for disposable use. This advantage is particularly beneficial not only for laboratory testing but also for practical material production in industrial settings, where frequent replacement of clogged devices and the use of multiple devices are often necessary.

## Data Availability

The data presented in this study are available from the corresponding author upon reasonable request.

## Supplementary Materials

The following supporting information can be downloaded at: https://www.mdpi.com/article/10.3390/mi16020132/s1, Figure S1: Schematic representation of the SE device with straight nozzles; Figure S2: SEM images of master molds and replicated PDMS nozzles; Figure S3: O/W droplet micrographs and size distributions from the device with triangular nozzles; Figure S4: An SEM image and size distribution of the polymeric microspheres; Figure S5: W/O droplet generation; Figure S6: Partial wetting of the triangular nozzles; Table S1: Shrinking factors; Video S1: Step emulsification in the SD mode; Video S2: Step emulsification in the LD mode.

## References

[1] Nan L., Zhang H., Weitz D.A., Shum H.C. (2024). Development and future of droplet microfluidics. Lab Chip.

[2] Kojabad A.A., Farzanehpour M., Galeh H.E.G., Dorostkar R., Jafarpour A., Bolandian M., Nodooshan M.M. (2021). Droplet digital PCR of viral DNA/RNA, current progress, challenges, and future perspectives. J. Med. Virol..

[3] Long F., Guo Y., Zhang Z., Wang J., Ren Y., Cheng Y., Xu G. (2023). Recent progress of droplet microfluidic emulsification based synthesis of functional microparticles. Glob. Chall..

[4] Kawakatsu T., Kikuchi Y., Nakajima M. (1997). Regular-sized cell creation in microchannel emulsification by visual microprocessing method. J. Am. Oil Chem. Soc..

[5] Shi Z., Lai X., Sun C., Zhang X., Zhang L., Pu Z., Wang R., Yu H., Li D. (2020). Step emulsification in microfluidic droplet generation: Mechanisms and structures. Chem. Commun..

[6] Liu Z., Duan C., Jiang S., Zhu C., Ma Y., Fu T. (2020). Microfluidic step emulsification techniques based on spontaneous transformation mechanism: A review. J. Ind. Eng. Chem..

[7] Dangla R., Kayi S.C., Baroud C.N. (2013). Droplet microfluidics driven by gradients of confinement. Proc. Natl. Acad. Sci. USA.

[8] Sahin S., Schroën K. (2015). Partitioned EDGE devices for high throughput production of monodisperse emulsion droplets with two distinct sizes. Lab Chip.

[9] Eggersdorfer M.L., Seybold H., Ofner A., Weitz D.A., Studart A.R. (2018). Wetting controls of droplet formation in step emulsification. Proc. Natl. Acad. Sci. USA.

[10] Amstad E., Chemama M., Eggersdorfer M., Arriaga L.R., Brenner M.P., Weitz D.A. (2016). Robust scalable high throughput production of monodisperse drops. Lab Chip.

[11] Kawakatsu T., Komori H., Nakajima M., Kikuchi Y., Yonemoto T. (1999). Production of monodispersed oil-in-water emulsion using crossflow-type silicon microchannel plate. J. Chem. Eng. J..

[12] Ofner A., Moore D.G., Rühs P.A., Schwendimann P., Eggersdorfer M., Amstad E., Weitz D.A., Studart A.R. (2017). High-throughput step emulsification for the production of functional materials using a glass microfluidic device. Macromol. Chem. Phys..

[13] Kobayashi I., Wada Y., Uemura K., Nakajima M. (2008). Generation of uniform drops via through-hole arrays micromachined in stainless-steel plates. Microfluid. Nanofluid..

[14] Kobayashi I., Hirose S., Katoh T., Zhang Y., Uemura K., Nakajima M. (2008). High-aspect-ratio through-hole array microfabricated in a PMMA plate for monodisperse emulsion production. Microsyst. Technol..

[15] Schuler F., Schwemmer F., Trotter M., Wadle S., Zengerle R., von Stetten F., Paust N. (2015). Centrifugal step emulsification applied for absolute quantification of nucleic acids by digital droplet RPA. Lab Chip.

[16] Zhan W., Liu Z., Jiang S., Zhu C., Ma Y., Fu T. (2022). Comparison of formation of bubbles and droplets in step-emulsification microfluidic devices. J. Ind. Eng. Chem..

[17] Xia Y., Whitesides G.M. (1998). Soft lithography. Angew. Chem. Int. Ed. Engl..

[18] Postek W., Kaminski T.S., Garstecki P. (2017). A passive microfluidic system based on step emulsification allows the generation of libraries of nanoliter-sized droplets from microliter droplets of varying and known concentrations of a sample. Lab Chip.

[19] Stolovicki E., Ziblat R., Weitz D.A. (2018). Throughput enhancement of parallel step emulsifier devices by shear-free and efficient nozzle clearance. Lab Chip.

[20] Håti A.G., Szymborski T.R., Steinacher M., Amstad E. (2018). Production of monodisperse droplets from viscous fluids. Lab Chip.

[21] de Rutte J.M., Koh J., Di Carlo D. (2019). Scalable high-throughput production of modular microgels for in situ assembly of microporous tissue scaffolds. Adv. Funct. Mater..

[22] Shin D.-C., Morimoto Y., Sawayama J., Miura S., Takeuchi S. (2019). Centrifuge-based step emulsification device for simple and fast generation of monodisperse picoliter droplets. Sens. Actuators B Chem..

[23] Schulz M., Probst S., Calabrese S., Homann A.R., Borst N., Weiss M., von Stetten F., Zengerle R., Paust N. (2020). Versatile tool for droplet generation in standard reaction tubes by centrifugal step emulsification. Molecules.

[24] Zhao S., Zhang Z., Hu F., Wu J., Peng N. (2021). Massive droplet generation for digital PCR via a smart step emulsification chip integrated in a reaction tube. Analyst.

[25] Zheng Y., Chen H., Lin X., Li M., Zhao Y., Shang L. (2023). Scalable production of biomedical microparticles via high-throughput microfluidic step emulsification. Small.

[26] Mittal N., Cohen C., Bibette J., Bremond N. (2014). Dynamics of step-emulsification: From a single to a collection of emulsion droplet generators. Phys. Fluids.

[27] Nalin F., Tirelli M.C., Garstecki P., Postek W., Costantini M. (2024). Tuna-step: Tunable parallelized step emulsification for the generation of droplets with dynamic volume control to 3D print functionally graded porous materials. Lab Chip.

[28] Eggersdorfer M.L., Zheng W., Nawar S., Mercandetti C., Ofner A., Leibacher I., Koehler S., Weitz D.A. (2017). Tandem emulsification for high-throughput production of double emulsions. Lab Chip.

[29] Opalski A.S., Makuch K., Derzsi L., Garstecki P. (2020). Split or slip—Passive generation of monodisperse double emulsions with cores of varying viscosity in microfluidic tandem step emulsification system. RSC Adv..

[30] Ji G., Kanno Y., Nisisako T. (2023). Microfluidic coupling of step emulsification and deterministic lateral displacement for producing satellite-free droplets and particles. Micromachines.

[31] Zheng C., Masui S., Kanno Y., Nisisako T. (2024). Microfluidic step emulsification with parallel nozzles on a vertical slit. Ind. Eng. Chem. Res..

[32] Nakashima T., Shimizu M., Kukizaki M. (2000). Particle control of emulsion by membrane emulsification and its applications. Particle control of emulsion by membrane emulsification and its applications. Adv. Drug Deliv. Rev..

[33] Wu W., Zhou S., Hu J., Wang G., Ding X., Gou T., Sun J., Zhang T., Mu Y. (2018). A thermosetting oil for droplet-based real-time monitoring of digital PCR and cell culture. Adv. Funct. Mater..

[34] Bauer W.-A.C., Fischlechner M., Abell C., Huck W.T.S. (2010). Hydrophilic PDMS microchannels for high-throughput formation of oil-in-water microdroplets and water-in-oil-in-water double emulsions. Lab Chip.

[35] Trantidou T., Elani Y., Parsons E., Ces O. (2017). Hydrophilic surface modification of PDMS for droplet microfluidics using a simple, quick, and robust method via PVA deposition. Microsys. Nanoeng..

[36] Ji G., Masui S., Kanno Y., Nisisako T. (2024). Upscaled production of satellite-free droplets: Step emulsification with deterministic lateral displacement. Micromachines.

[37] Yao M., Fang J. (2012). Hydrophilic PEO-PDMS for microfluidic applications. J. Micromech. Microeng..

[38] Lee J.N., Park C., Whitesides G.M. (2003). Solvent compatibility of poly(dimethylsiloxane)-based microfluidic devices. Anal. Chem..

[39] Kim B.-Y., Hong L.-Y., Chun Y.-M., Kim D.-P., Lee C.-S. (2009). Solvent resistant PDMS microfluidic devices with hybrid inorganic/organic polymer coatings. Adv. Funct. Mater..

[40] Nisisako T. (2016). Recent advances in microfluidic production of Janus droplets and particles. Curr. Opin. Colloid Interface Sci..

