# Supplementary material for: Production of Monodisperse Oil-in-Water Droplets and Polymeric Microspheres Below 20 μm Using a PDMS-Based Step Emulsification Device
Source: Micromachines (Basel). 2025 Jan 24;16(2):132. doi: 10.3390/mi16020132 (PMC11857107; doi:10.3390/mi16020132)
Supplement: Supplementary file 1 [file micromachines-16-00132-s001.zip › micromachines-3427090 Supplementary Materials.pdf]

Supplementary Material

Production of Monodisperse Oil-in-Water Droplets and Polymeric Microspheres Below 20 μm Using a PDMS-Based Step Emulsification Device

Naotomo Tottori [1], Seungman Choi [1] and Takasi Nisisako [2,*]

[1] Department of Mechanical Engineering, School of Engineering, Institute of Science Tokyo, Tokyo 152-8550, Japan
[2] Laboratory for Future Interdisciplinary Research of Science and Technology (FIRST), Institute of Integrated Research, Institute of Science Tokyo, R2-9, 4259 Nagatsuta-cho, Midori-ku, Yokohama 226-8501, Japan
* Correspondence: nisisako.t.aa@m.titech.ac.jp; Tel.: +81-45-924-5092

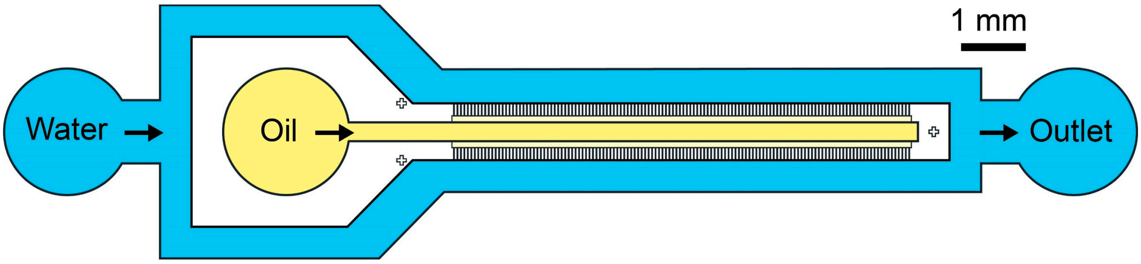

Figure S1. Schematic representation of the overall layout of the polydimethylsiloxane (PDMS)-based step-emulsification (SE) device with 264 straight nozzles and an upstream plateau.

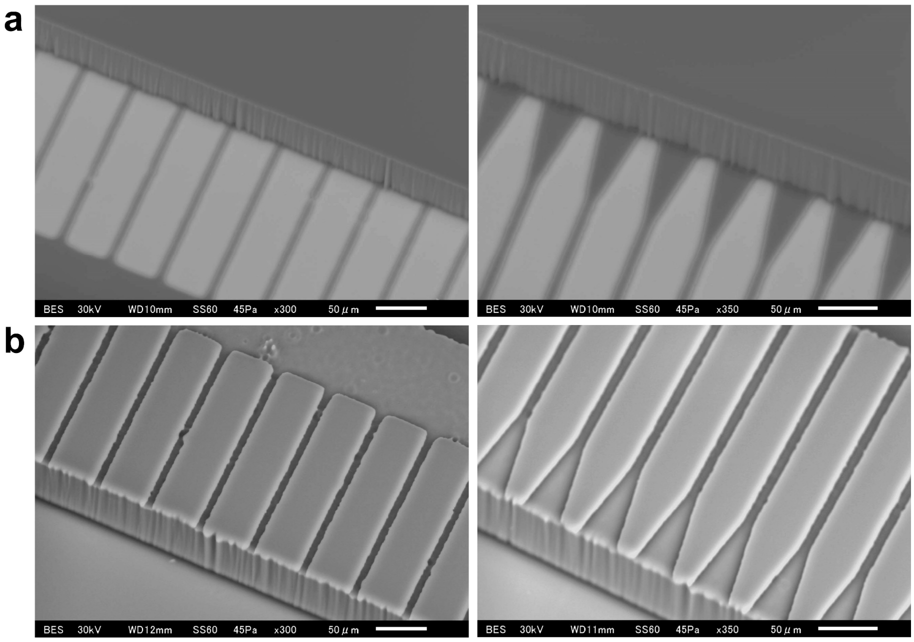

Figure S2. Scanning electron microscopy (SEM) images of (a) master molds and (b) microchannels replicated in PDMS for the SE devices with straight nozzles (left) and triangular nozzles (right). Scale bars: 50 μm.

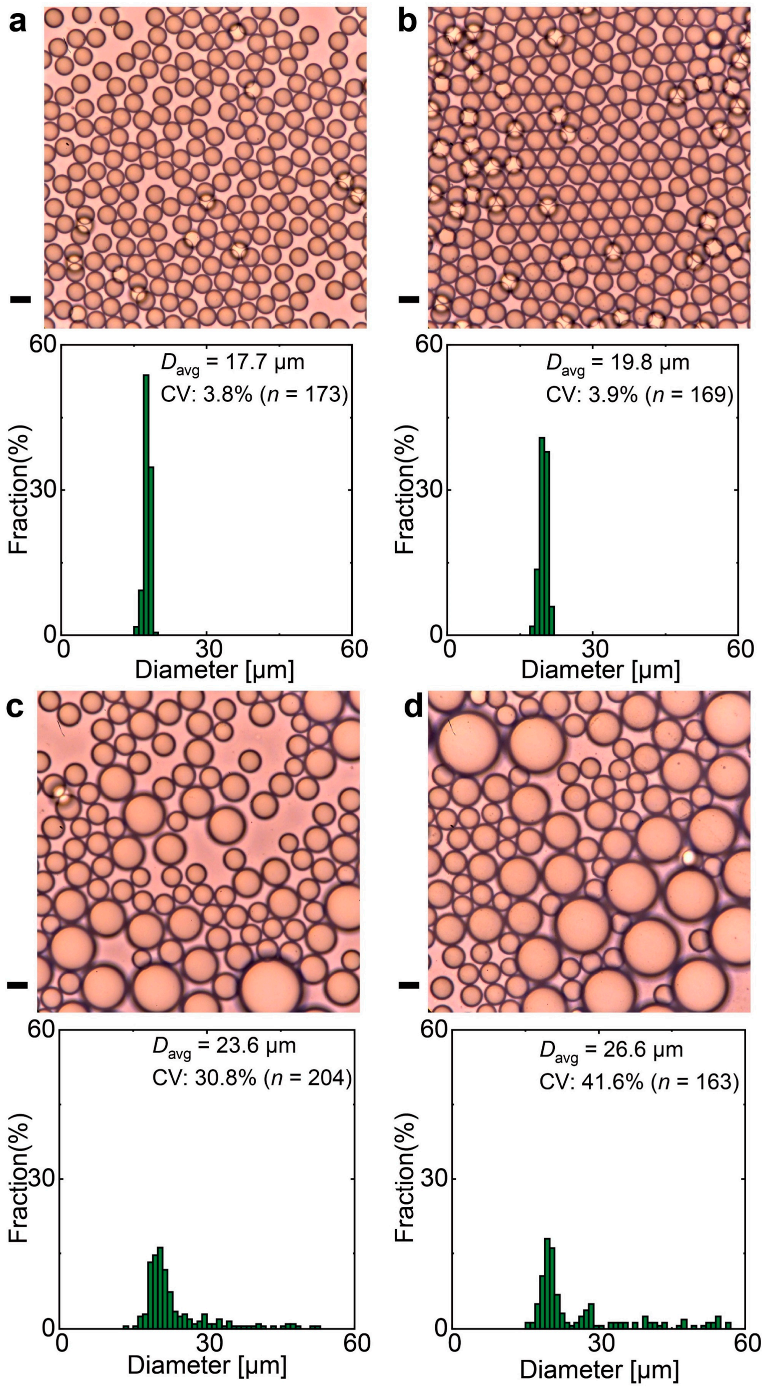

Figure S3. Oil-in-water (O/W) droplet micrographs and size distributions from the device with triangular nozzles at $Q_c = 5.0$ mL h^{-1} and Q_d = (a) 0.3 mL h^{-1}, (b) 0.5 mL h^{-1}, (c), 0.6 mL h^{-1}, and (d) 1.0 mL h^{-1}. Scale bars: 20 μm.

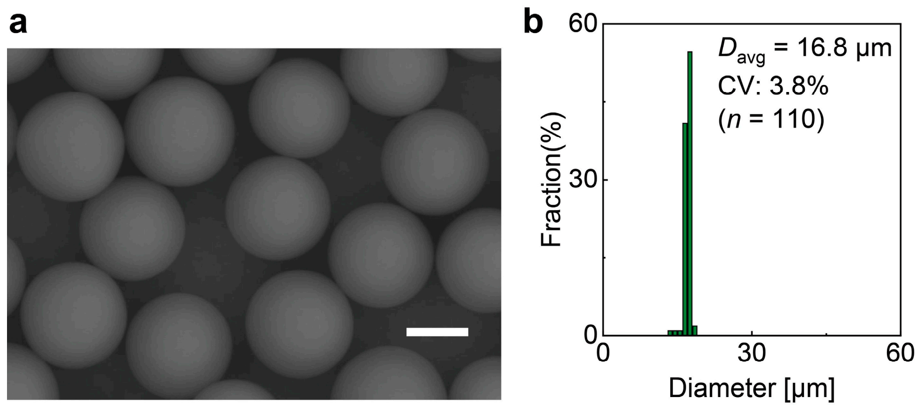

Figure S4. (a) An SEM image and (b) size distribution of the polymeric microspheres prepared at Q_c = 5.0 mL h^{-1} and Q_d = 0.3 mL h^{-1}. Scale bar: 10 μm.

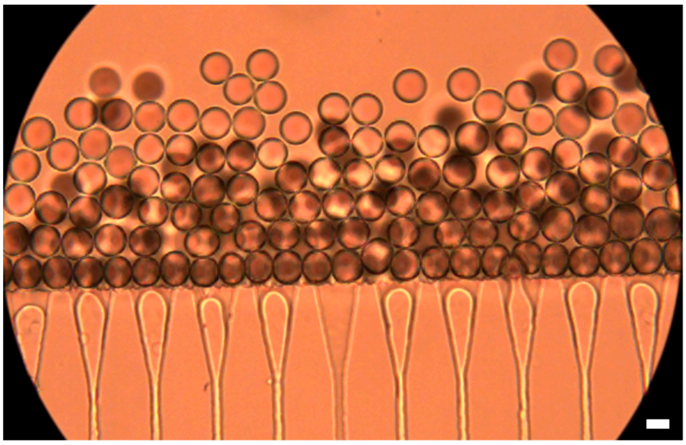

Figure S5. Water-in-oil (W/O) droplet generation using the SE device with restored hydrophobic PDMS nozzles, operated at Q_c = 1.0 mL h^{-1} and Q_d = 0.05 mL h^{-1}. Scale bar: 20 μm.

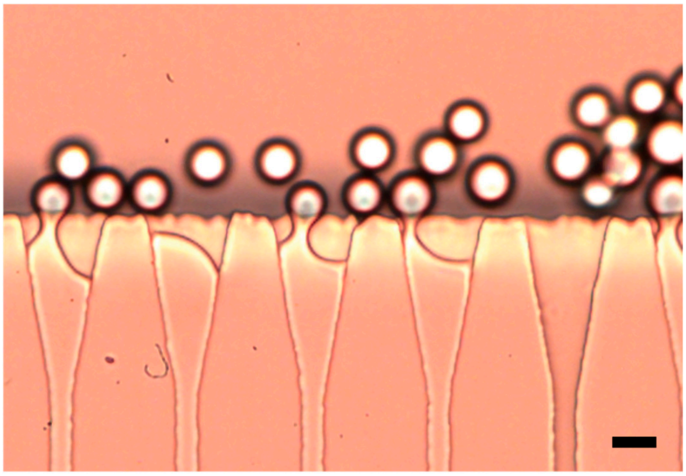

Figure S6. O/W droplet generation at triangular SE nozzles with partial wetting of PDMS walls by HDDA, operated at Q_c = 5.0 mL h^{-1} and Q_d = 0.5 mL h^{-1}. Scale bar: 20 μm.

Table S1. Initial droplet diameters (D_d), resulting particle diameters (D_p), and corresponding shrinking factors (D_p/D_d) obtained from various experiments using the same fluid combination.

#	D_d [μm]	D_p [μm]	D_p / D_d [-]	Source
1	17.0	16.1	0.947	Figs. 5a, 7
2	17.7	16.8	0.949	Figs. S3a, S4b
3	58.0	55.0	0.948	Ref. [30]
4	68.0	64.0	0.941	Ref. [31]

Supplementary Video Captions

Video S1. Step emulsification in the SD mode at Q_c = 5.0 mL h^{-1} and Q_d = 0.1 mL h^{-1}, recorded using a high-speed video camera at 500 fps.

Video S2. Step emulsification in the LD mode at Q_c = 5.0 mL h^{-1} and Q_d = 0.7 mL h^{-1}, recorded using a high-speed video camera at 500 fps.

All files are in MEPG-4 format.
